# Safety and Feasibility of On-the-Table Pharmacomechanical Lysis for Acute Intermediate-Risk Pulmonary Embolism: The RESCUE-II Study

**DOI:** 10.1016/j.jacadv.2025.101789

**Published:** 2025-05-19

**Authors:** Vladimir Lakhter, Christian Bichard, Kenneth Ouriel, Brian Firth, Parth Rali, Riyaz Bashir

**Affiliations:** a Division of Cardiovascular Diseases, Temple University Hospital, Philadelphia, Pennsylvania, USA; b Department of Internal Medicine, Temple University Hospital, Philadelphia, Pennsylvania, USA; c North American Science Associates, New York, New York, USA; d Thrombolex, New Britain, Pennsylvania, USA; e Department of Thoracic Medicine and Surgery, Temple University Hospital, Philadelphia, Pennsylvania, USA

**Keywords:** catheter-directed thrombolysis, pharmacomechanical lysis, pulmonary artery obstruction, pulmonary embolism

## Abstract

**Background:**

Acute pulmonary embolism (PE) is a leading cause of morbidity and mortality. Pharmacomechanical lysis (PML) with the Bashir endovascular catheter has been shown to reduce the right ventricular/left ventricular (RV/LV) ratio in patients with intermediate-risk (IR) PE. Nevertheless, the original protocol required a 5-hour postprocedural infusion of recombinant tissue plasminogen activator (r-tPA) and intensive care unit monitoring.

**Objectives:**

The RESCUE-II (Safety and Feasibility of On-The-Table Pharmacomechanical Lysis for Acute Intermediate-Risk Pulmonary Embolism) study aimed to evaluate the safety and efficacy of on-the-table PML using bolus-only r-tPA, without postprocedural infusion, in patients with IR-PE.

**Methods:**

In this single-center, prospective study, symptomatic patients with IR-PE (computed tomographic–derived RV/LV ratio ≥0.9) were treated with bolus-only r-tPA via the Bashir catheter (4 mg per lung, 8 mg total for bilateral PE). The primary efficacy endpoint was the change in RV/LV ratio at 48 hours, and the primary safety endpoint was major bleeding within 72 hours.

**Results:**

Nine patients were enrolled and successfully treated. The median procedure time was 39 ± 13.4 minutes. At 48 hours, the mean RV/LV ratio decreased from 1.66 ± 0.56 to 1.27 ± 0.41 (*P* = 0.0001), and pulmonary artery obstruction, measured by the Refined Modified Miller index, reduced by 29.2%. There were no major bleeding events. One patient had a minor access site hematoma, managed with manual compression.

**Conclusions:**

On-the-table PML using the Bashir catheter effectively reduced RV/LV ratio and PA obstruction. The procedure was safe, with no major bleeding complications, and offers a rapid, cost-effective treatment option for patients with acute IR-PE.

Acute pulmonary embolism (PE) is a major cause of morbidity and mortality in the United States and around the world.^1^^,^^2^ Although most patients with intermediate-risk PE may do well with therapeutic anticoagulation alone, there remains a 30-day mortality rate of 10% associated with this strategy.^3^^,^^4^ For this reason, an increasing number of intermediate-risk PE patients are now undergoing adjunctive endovascular treatments.^5^ Two of the most used interventional strategies are catheter-based mechanical thrombectomy (MT) and catheter-directed thrombolysis (CDT). Several ongoing randomized trials are evaluating whether adjunctive catheter-directed therapies, in addition to therapeutic anticoagulation, can improve the outcomes of patients with intermediate-risk PE.^6^, ^7^, ^8^

More recently, a novel approach of catheter-directed pharmacomechanical lysis (PML) using a Bashir endovascular catheter has been developed to treat patients with acute PE.^9^ This approach entails a combination of mechanical thrombus fragmentation, creating a sizeable endoluminal channel through the blood clot to restore blood flow and expose a large surface area of the clot to exogenously administered recombinant tissue plasminogen activator (r-tPA) and endogenous fibrinolytics. Small doses of r-tPA are then delivered via hand-injected pulse sprays directly into the body of the clot, where it remains active for several hours. This approach was shown to be both safe and effective in the pivotal RESCUE trial despite using a significantly smaller r-tPA dose (7 mg per lung) than in previous trials.^10^

The treatment protocol used in the RESCUE trial required a 5-hour r-tPA infusion after an initial 2 mg r-tPA bolus into each affected lung. This necessitated an intensive care unit (ICU) admission for the duration of the infusion. Bench-top testing has shown that the amount of active r-tPA is reduced by approximately 50% when infused in the standard manner due to the adsorption of r-tPA to the infusion bag and IV tubing.^11^^,^^12^ Therefore, we designed the present trial to administer 4 mg of r-tPA into each lung, by pulse spray only, for a total of 8 mg of r-tPA for bilateral PE, compared to the total dose of 14 mg administered in the pivotal RESCUE study. This strategy would allow the procedure to be completed on the table, thereby rendering ICU admission unnecessary. In this study, we sought to evaluate the safety and feasibility of bolus-only “on-the-table” r-tPA administration via the Bashir endovascular catheter for treating patients with acute intermediate-risk PE.

## Methods

### Study design

The RESCUE-II (Safety and Feasibility of On-The-Table Pharmaco-mechanical Lysis for Acute Intermediate-Risk Pulmonary Embolism) study is an investigator-initiated, prospective, single-center, single-arm study that assessed the feasibility of on-the-table PML using the Bashir endovascular catheter for the treatment of patients with acute intermediate-risk PE, without subsequent r-tPA infusion. The Department of Health of the Commonwealth of Pennsylvania sponsored the study. Institutional Review Board approval was obtained, as was written informed consent from each patient.

### Study population

The study was designed to enroll patients 18 to 75 years of age who were diagnosed with acute intermediate-risk PE. Patients were considered for the study if they had a filling defect in at least 1 main or lobar pulmonary artery (PA) on contrast-enhanced chest CT angiogram (CTA), right ventricular (RV) to left ventricular diameter (LV) ratio ≥0.9, and symptom duration ≤14 days. Major exclusion criteria for this study included cerebrovascular accident or transient ischemic attack within 1 year; head trauma or other active intracranial or intraspinal disease within 1 year; COVID-19 infection within the preceding 2 months; recent (within 1 month) or active bleeding from a major organ; hematocrit <30%; platelets <100,000/μL; international normalized ratio >1.5; serum creatinine >2 mg/dL; and systolic blood pressure <90 mm Hg for >15 minutes; and any vasopressor or inotropic support. The complete list of inclusion and exclusion criteria is listed in the Supplemental Table 1.

### Device description and procedural technique

The 0.035-inch guidewire-compatible Bashir endovascular catheter is an 8-F device approved for localized infusion of therapeutic substances into the vasculature, including the PA. The Bashir catheter has been previously described in detail.^10^ The catheter's expandable infusion basket comprises 6 individual nitinol-reinforced infusion limbs arranged in a helical manner, each containing 8 laser-drilled holes that allow delivery of therapeutic agents, including r-tPA. Initial basket expansion within the clot provides for rapid restoration of native blood flow and initiation of an endogenous thrombolytic process. After basket expansion, r-tPA is administered via each of the 6 mini-infusion catheters that comprise the infusion basket. In the present study, r-tPA was administered as a high pressure hand bolus only without any follow-up infusion.

Venous access, using ultrasound guidance and a micropuncture needle, was mandated via either the femoral or internal jugular veins. After placement of a short 8F sheath, right heart catheterization was performed. Following completion of a hemodynamic assessment, the short sheath was exchanged for a long 8F sheath (usually 70 cm in length from a femoral approach and 45 cm from an internal jugular approach). The Bashir catheter was then advanced over a 0.035-inch guidewire through a long 8F sheath and positioned such that the infusion basket was placed within the thrombus in the PA (main PA, interlobar artery, or truncus anterior). Once in place, the sheath was retracted, and the infusion basket was expanded under fluoroscopy by retracting the actuator on the handle. Following basket expansion, a bolus of dilute r-tPA (1 mg r-tPA in 10 mL 0.9% saline) was hand-injected. After administration of each r-tPA bolus, the basket was collapsed, then re-expanded, and followed by a bolus of 10 mL of 0.9% saline. This sequence was repeated 4 times within each affected PA. Therefore, a total of 4 mgs of r-tPA was delivered to each PA, and the infusion basket was expanded and collapsed 8 times.

**Figure 1:**
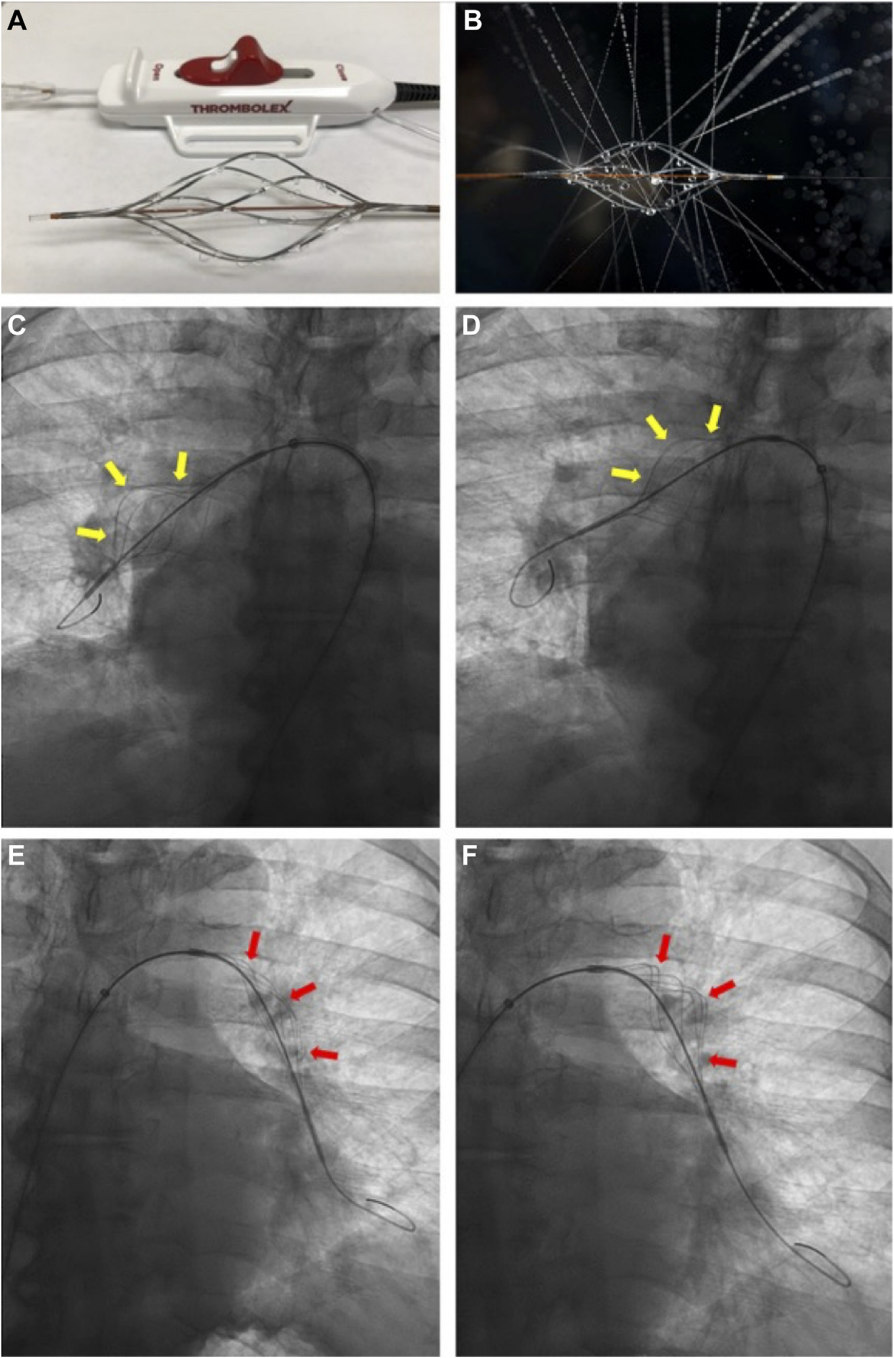
Bashir Catheter Positioning During Pharmacomechanical Catheter-Directed Thrombolysis Bashir catheter has an expandable basket which is controlled by a red actuator (A); upon basket expansion, r-tPA can be delivered intravascularly via each of the 6 struts that make up the expansion basket (B). Bashir catheter expansion within the right-sided pulmonary artery over an 0.035-inch wire. The catheter is expanded within the mid-distal interlobar artery (C; yellow arrows) and then within the main and proximal interlobar pulmonary artery (D; yellow arrows). The 7-F 70 cm sheath is then pulled back and repositioned into the left-sided pulmonary artery. Bashir catheter is expanded within the mid-distal (E; red arrows) and proximal-mid (F; red arrows) interlobar artery; each expansion is followed by a high-pressure pulse spray by a hand-injection of dilute r-tPA. r-tPA = recombinant tissue plasminogen activator.

After treatment within a particular lung, the Bashir catheter was retracted into the 8F sheath, and an 0.035-inch wire was manipulated into the contralateral PA. Once the guidewire had been positioned within the contralateral PA, the infusion catheter was readvanced and 4 mg of tPA was delivered using the same protocol as described above. Authors commonly treat the right PA first since repositioning the catheter from the right PA into the left PA is easier than the reverse (Figure 1).

Once both PAs were treated, the PA saturations and PA pressures were rechecked. After this, the Bashir catheter and the long sheath were removed, and hemostasis was obtained using manual compression. Therapeutic anticoagulation using unfractionated heparin or enoxaparin was restarted about 1 hour after achieving hemostasis. During the procedure, for patients in whom unfractionated heparin had been used prior to the procedure, additional boluses of intravenous heparin were administered to maintain the activated clotting time above 200 seconds. For those patients in whom enoxaparin had been used to maintain therapeutic anticoagulation prior to the procedure, no further intraprocedural anticoagulation was administered if the most recent enoxaparin dose had been given within the previous 8 hours. Patients were usually transitioned to an oral anticoagulant the day after the procedure.

### Efficacy and safety endpoints

The study's primary efficacy endpoint was the reduction in the CTA-derived RV/LV ratio at 48 hours after procedural completion. The primary safety endpoint was the rate of major bleeds at 72 hours (International Society on Thrombosis and Haemostasis criteria). The secondary endpoints included the following: changes in PA obstruction as defined by the Refined Modified Miller Index (RMMI), as measured on contrast-enhanced chest CTA within 48 hours after the completion of the r-tPA bolus administration compared to baseline, measured by the independent core lab; reduction in segmental PA total and subtotal occlusions at 48 hours after on-the-table PML using the core laboratory assessment of CTA as previously described^10^^,^^13^; all-cause mortality from hospital discharge through 30-day follow-up; and serious adverse events (AEs) through 30-day follow-up. The complete list of primary and secondary endpoints is listed in Supplemental Table 2.

A dedicated imaging core laboratory measured changes in RV/LV diameter ratio and PA obstruction index using anonymized chest computed tomographic angiographic studies at baseline and 48 hours after treatment. The RV/LV diameter ratio was assessed using the reformatted 4-chamber view.^14^ PA obstruction was calculated using the RMMI, a modified Miller scoring system refinement.^15^

As was previously reported, the RMMI assesses the degree of obstruction in 10 segmental arteries in the right lung and 10 in the left lung and assigns a score of 0 (no obstruction), 0.5 (1%-33% obstruction), 1 (34%-66% obstruction), 1.5 (67%-99% obstruction), or 2 (total occlusion) to each artery. A cumulative score is calculated by adding the scores for all arteries. The total scores range from 0 to 40 (20 per side). The proximal PA branch scores are calculated based on the number of segmental arteries that arise from that proximal artery as long as the highest possible scores are ascribed to each branch. These proximal branches included the 2 main PAs, 2 upper lobe truncus branches, and 2 lower lobe interlobar arteries. A data and safety monitoring board and clinical events committee adjudicated all AEs.

### Data analysis

Descriptive statistics were used to summarize the data collected. Paired *t*-test was used to compare continuous variables from baseline to post-PML. All *P* values were 2-sided and were considered significant if <0.05 (our estimates were not adjusted for multiple comparisons and should be interpreted with caution). The data were only analyzed once at the completion of enrollment of all patients. The clinical adjudication committee adjudicated the clinical events. The imaging studies were evaluated in a blinded fashion by an independent Core Laboratory, NAMSA Inc (Syntactx). The study sponsors were not involved in the design or data analysis, or interpretation of this study. PharmaLex Inc performed statistical analyses.

## Results

### Baseline characteristics

A total of 10 patients were screened and consented between January 2024 and July 2024, of which 9 were qualified, enrolled, and treated in our institution. All patients had an RV/LV ratio >0.9 and met the criteria for intermediate-risk PE based on the European Society of Cardiology guidelines,^3^ with all of the patients being in the high intermediate-risk category with elevation of troponin or brain natriuretic peptide levels. The mean age was 68.3 ± 11 years, with a mean body mass index of 32.3 ± 6.9 kg/m^2^; 55.6% of patients were male. All patients had bilateral PE. A complete list of patient characteristics is provided in Table 1.

**Table 1:** Baseline Demographic and Clinical Characteristics (n = 9)

Clinical Characteristic	
Age (y)	68.3 ± 11
Body mass index, kg/m^2^	32.3 ± 6.9
Male	5 (55.6%)
Female	4 (44.4%)
White/non-Hispanic	1 (11.1%)
Black/African American	6 (66.7%)
Hispanic	1 (11.1%)
Other	1 (11.1%)
BNP^a^, pg/mL	312 ± 450
Troponin, ng/mL	0.57 ± 0.78
History of cancer	2 (22.2%)
Diabetes mellitus	3 (33.3%)
Previous deep vein thrombosis	2 (22.2%)
History of pulmonary embolism	2 (22.2%)
Bilateral pulmonary embolism	9/9 (100%)
Elevated troponin or BNP	9/9 (100%)
Elevated troponin	9/9 (100%)
Elevated BNP	5/9 (55.6%)

Values are mean ± SD or n (%).

a Brain natriuretic peptide.

### Procedural characteristics

A total of 9 Bashir catheters were placed in the 9 enrolled patients. In all 9 patients, a single Bashir catheter was used to treat both the right and left PAs. The total dose of r-tPA was 8 mg in all patients; 4 mg r-tPA was administered into each lung in all patients. All devices (100%) were successfully placed. The median total procedure time was 39 ± 13.4 minutes (Table 2). The total treatment time from insertion to the removal of the Bashir catheter was 17 ± 3 minutes. Two patients came from an ICU and returned to the same unit because of bed availability. None of the patients required an upgrade to ICU after the procedure if they initially presented to the cath lab from a non-ICU bed.

**Table 2:** Procedural Characteristics (n = 9)

Procedural Characteristic	
Procedural success	9/9 (100%)
Total r-tPA dose, mg	8
Total procedure time in minutes (median)	39 ± 13.4
Treatment time in minutes (median)^a^	17 ± 3.3
Number of patients where one device was used	9/9 (100%)

Values are n (%) or mean ± SD.

r-tPA = recombinant tissue plasminogen activator.

a Bashir catheter total dwell time.

### Efficacy outcomes

**Figure 2:**
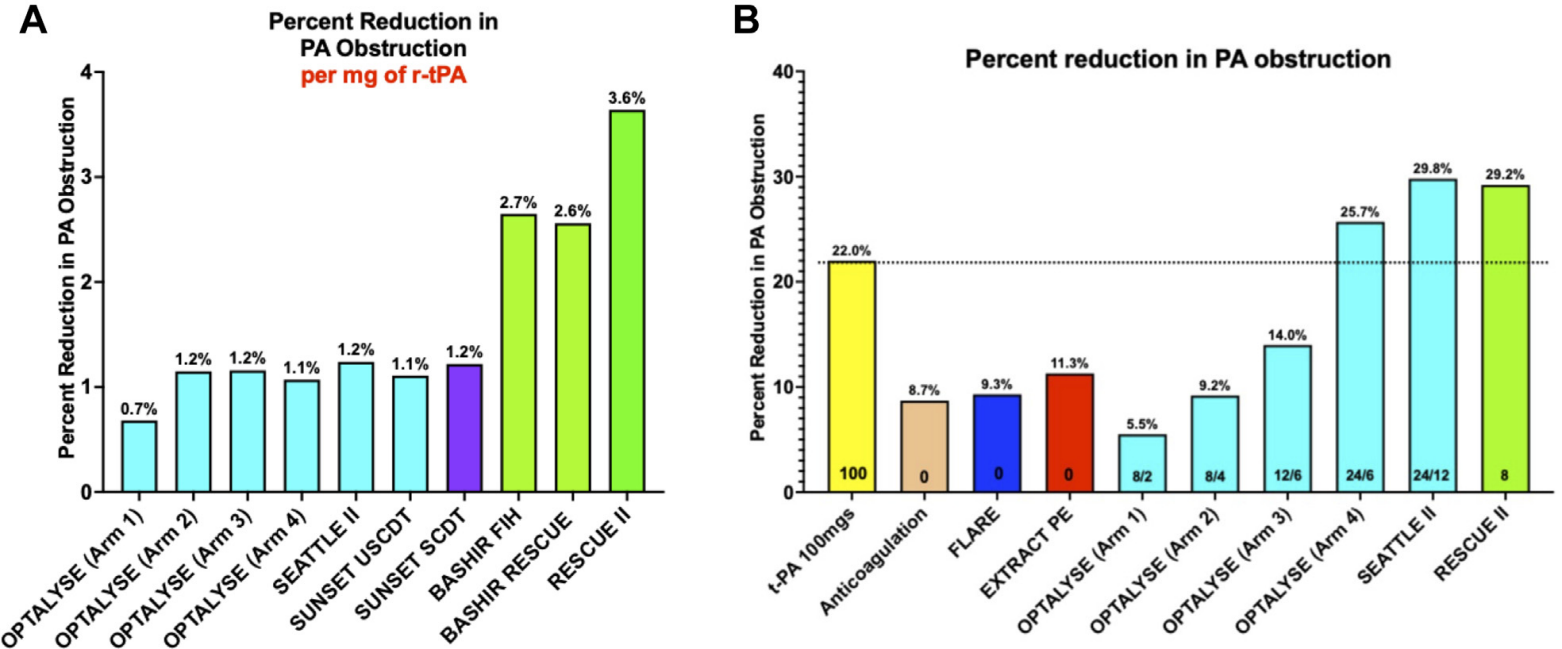
Comparison of Thrombolytic Efficiency and Reduction in PA Obstruction Across Contemporary Acute PE Trials Thrombolytic efficiency expressed in terms of percent reduction in PA obstruction per mg of r-tPA was shown to be the greatest in the RESCUE II study; by comparison, thrombolytic efficiency seen in CDT using single lumen infusion catheters was about a third of what was seen in RESCUE-II (Safety and Feasibility of On-The-Table Pharmaco-mechanical Lysis for Acute Intermediate-Risk Pulmonary Embolism) (A). The percent reduction in PA obstruction after PML with 8 mg of r-tPA used in the RESCUE-II study was very similar to that seen after CDT with 24 mg of r-tPA used in the SEATTLE II and OPTALYSE (A Randomized Trial of the Optimum Duration of Acoustic Pulse Thrombolysis Procedure in Acute Intermediate-Risk Pulmonary Embolism: The OPTALYSE PE trial) (Arm 4) trials. The percent reduction was also approximately 3 times as great as that seen in the FLARE (A Prospective, Single-Arm, Multicenter Trial of Catheter-Directed Mechanical Thrombectomy for Intermediate-Risk Acute Pulmonary Embolism: The FLARE Study) and EXTRACT PE (Indigo Aspiration System for Treatment of Pulmonary Embolism: Results of the EXTRACT-PE Trial) pivotal trials using percutaneous mechanical thrombectomy (B). CDT = catheter-directed thrombolysis; PA = pulmonary artery; PE = pulmonary embolism; PML = pharmacomechanical lysis; other abbreviation as in Figure 1.

At 48 hours after PML, the mean RV/LV diameter ratio decreased from 1.66 ± 0.56 to 1.27 ± 0.41 (*P* = 0.0001), a mean reduction of 0.39 ± 0.31 (22.3% reduction) (Central Illustration A, Table 3). Mean total PA obstruction (RMMI score) decreased from 23.9 ± 3.3 to 16.9 ± 3.7 (*P* = 0.0001), a mean reduction of 6.9 ± 3.1 (95% CI: 4.6-9.3; 29.2%) (Central Illustration B, Table 3). The percentage of segmental PA branches with a total or subtotal occlusion decreased from 31.1% to 13.9% (*P* < 0.0001; 55.4% reduction). This reduction was also noted in the proximal PA branches (main PA, interlobar PA, or a basal lower lobe trunk) with a reduction in the number of total or subtotal occlusions, from 29.6% to 13.0% (*P* = 0.0039; a 56.3% reduction). The lytic effect was seen not only within the branches near the infusion basket but also within segmental branches that were anatomically remote from the site of r-tPA infusion (Table 4). On a per-patient basis, the average number of segmental arteries that were totally or subtotally occluded decreased from a baseline of 6.2 ± 5.8 to 2.8 ± 3.1 at 48 hours, a 59.9% reduction (*P* = 0.044). The average number of proximal branches that were totally or subtotally occluded decreased from a baseline of 1.8 ± 1.1 to 0.8 ± 1.1 at 48 hours, a 65.6% reduction (*P* = 0.0028). We also measured the reduction in percent PA obstruction per milligram of r-tPA administered; the reduction in RMMI score was 3.6% per milligram of r-tPA (Figure 2A).

**Central Illustration:**
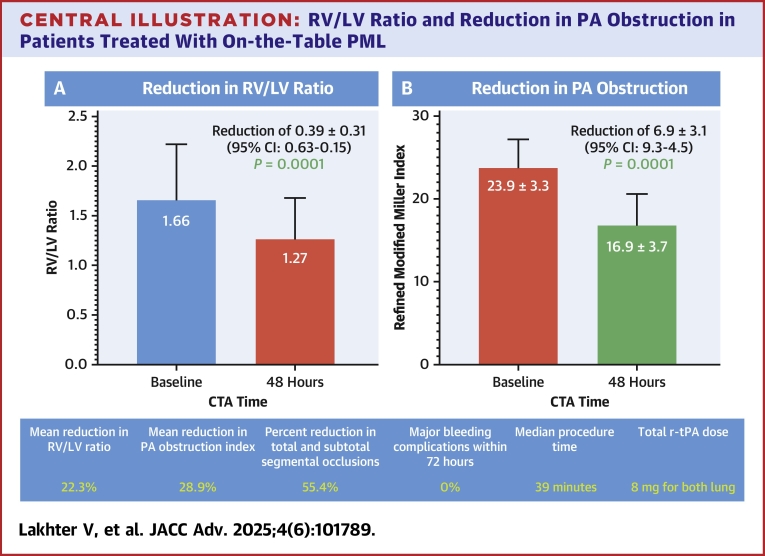
RV/LV Ratio and Reduction in PA Obstruction in Patients Treated With On-the-Table PML CTA-based RV/LV ratio at 48 hours after pharmacomechanical catheter-directed thrombolysis decreased from a baseline of 1.66 down to 1.27 (22.3% reduction) (A). Pulmonary artery obstruction based on the Refined Modified Miller Index decreased from 23.9 to 16.9 (28.9% reduction) 48 hours after treatment (B). LV = left ventricular; RV = right ventricular; other abbreviations as in Figures 1 and 2.

**Table 3:** Efficacy Outcomes (n = 9)

	Baseline	Post-PML	Absolute Difference	*P* Value^a^
RV/LV diameter ratio by CTA^b^	1.66 ± 0.56	1.27 ± 0.41	0.39 ± 0.31	0.0001
Refined modified Miller Index	23.9 ± 3.3	16.9 ± 3.7	6.9 ± 3.1	0.0001
Baseline percentage of total and subtotal occlusions (proximal arteries)^c^	29.6%	13.0%	16.6% (56.1% relative reduction)	<0.0001
Number of proximal pulmonary artery occlusions (vessels per patient)	1.8 ± 1.1	0.8 ± 1.1	1.0 ± 0.71	0.0028
Baseline percentage of total and subtotal occlusions (segmental arteries)	31.1%	13.9%	17.2% (55.4% relative reduction)	0.004
Number of segmental pulmonary artery occlusions (vessels per patient)	6.2 ± 5.8	2.8 ± 3.1	3.4 ± 4.3	0.044
Systolic PA pressure, mm Hg^d^	52 ± 14.4	48.3 ± 10.5	−3.2 ± 9.4	0.33
Cardiac output, L/min	4.4 ± 1.1	4.2 ± 1.1	−0.15 ± 0.42	0.31
Cardiac index, L/min/m^2^	2.3 ± 0.48	2.0 ± 0.41	−0.23 ± 0.35	0.093

Values are mean ± or %.

RV/LV = right ventricular/left ventricular.

a Our estimates were not adjusted for multiple comparisons and should be interpreted with caution.

b Computed tomography angiography.

c Proximal pulmonary arteries = main PA, interlobar PA, or a basal lower lobe trunk.

d Pulmonary angiography.

**Table 4:** Segmental Artery Occlusion Analysis: Change in Number of Totally and Subtotally Occluded Segmental Arteries From Baseline to Post-PML

	Baseline (Number of Arteries)	Post-PML (Number of Arteries)	All Arteries	Percent Reduction (%)
Total and subtotal occlusions	56	25	180	55.4
RUL apical	3	2	9	33.3
RUL posterior	3	0	9	100
RUL anterior	2	2	9	0
RML medial	3	2	9	33.3
RML lateral	3	1	9	66.7
RLL superior	4	1	9	75
RLL posterior basal	4	3	9	25
RLL lateral basal	5	3	9	40
RLL anterior basal	4	2	9	50
RLL medial	4	2	9	50
LUL apical	4	1	9	75
LUL posterior	3	0	9	100
LUL anterior	2	1	9	50
LLL (lingula) superior lingula	1	0	9	100
LLL (lingula) inferior lingula	1	0	9	100
LLL superior	3	1	9	66.7
LLL posterior basal	3	1	9	66.7
LLL lateral basal	2	1	9	50
LLL anterior basal	1	1	9	0
LLL medial basal	1	1	9	0

LLL = left lower lobe; LUL = left upper lobe; PML = pharmacomechanical lysis; RLL = right lower lobe; RML = right middle lobe; RUL = right upper lobe.

### Safety outcomes

There were no major bleeding complications within 72 hours of r-tPA administration by the International Society on Thrombosis and Haemostasis criteria. There were no deaths or serious AEs through 30-day follow-up. One patient developed clinically relevant nonmajor bleeding due to an access site venous ooze, which was managed successfully with manual compression alone. The complete list of safety outcomes is listed in Table 5.

**Table 5:** Safety Outcomes (n = 9)

Outcome	Percentage
Major bleeding within 72 h (ISTH criteria)^a^	0 (0%)
Major device-related AEs^b^	0 (0%)
Clinically relevant nonmajor bleeding	1/9 (11.1%)
All-cause mortality through 30 d	0 (0%)
Serious AEs through 30 d	0 (0%)
Recurrent PE through 30-d follow-up^c^	0 (0%)
Patients requiring ICU upgrade after procedure^d^	0 (0%)

Values are n (%).

a International Society on Thrombosis and Haemostasis criteria.

b Adverse events.

c Pulmonary embolism.

d Intensive care unit.

## Discussion

In the present study, we evaluated the feasibility and safety of on-the-table bolus-only r-tPA, without postprocedural infusion, for treating patients with intermediate-risk PE. Our study showed that on-the-table r-tPA administration by high pressure bolus pulse sprays delivered by hand into the PAs, using the Bashir endovascular catheter resulted in: 1) a significant reduction in the RV/LV ratio at 48 hours compared to baseline; 2) significantly reduced the degree of PA obstruction and the number of total and subtotal segmental and main pulmonary arterial occlusions; and 3) the procedure was completed on the table in 39 minutes, without any major bleeding and without the need for postprocedural ICU admission.

We observed a 22.3% reduction (an absolute reduction of 0.39) in the RV/LV ratio at 48 hours among patients treated with on-the-table bolus-only r-tPA protocol. Although the RV/LV ratio was 1.27 after treatment with PML, the starting RV/LV ratio in our patient cohort was 1.66, which is higher than in other contemporary trials. Nevertheless, the percent reduction in RV/LV ratio compares favorably with the reduction observed in other contemporary PE trials, including the recently published randomized controlled PEERLESS (Large-bore Mechanical Thrombectomy Versus Catheter-Directed Thrombolysis in the Management of Intermediate-risk Pulmonary Embolism) trial^16^, ^17^, ^18^ in which the CDT arm had a 22.9% reduction while the thrombectomy arm had a 25.2% reduction of the RV/LV ratio. This effect size was seen even though the average r-tPA dose in the PEERLESS CDT arm was 16 mg compared with the 8 mg dose used in the RESCUE-II study. The ability to produce such a reduction in RV size with half the dose of r-tPA and without a postprocedural infusion could represent a significant advance in the catheter-based treatment of acute PE.

There was a 29.2% reduction in PA obstruction index 48 hours after completion of the bolus-only protocol. This degree of reduction is similar to that seen in the SEATTLE-II (A Prospective, Single-Arm, Multicenter Trial of Ultrasound-Facilitated Catheter-Directed, Low-Dose Fibrinolysis for Acute Massive and Submassive Pulmonary Embolism) study,^17^ in which 24 mgs of r-tPA were used over 12 hours as opposed to 8 mgs used in the present study for bilateral acute PE. This reduction was substantially greater than that observed with other contemporary MT technologies that have reported this endpoint using an independent core laboratory (Figure 2B).^19^^,^^20^

There was a significant decrease in total and subtotal occlusions at both the proximal PA and segmental PA levels following the on-the-table PML. A similar effect size was previously observed following a 5-hour r-tPA infusion protocol in the RESCUE trial.^10^ Although the r-tPA bolus was administered using a Bashir catheter positioned at a proximal location (main PA and interlobar artery), the thrombolytic effect was seen throughout most of the PA segments in both lungs (Table 4). This finding is likely due to factors such as recirculation of nonthrombus bound r-tPA and activation of endogenous thrombolytics like pro-urokinase. The effect of r-tPA on total and subtotal occlusions in segmental arteries with the PML approach may be a potential advantage over approaches using large-bore MT devices that may not extract fragmented thrombus from the distal segmental arteries effectively.^21^ Given that these segmental arteries feed the alveolar capillary bed, where alveolar gas exchange occurs, persistent distal obstruction of the pulmonary arteries may contribute to decreased pulmonary venous blood volume, which has been shown to be a predictor of late mortality, or the development of chronic thromboembolic pulmonary vascular disease.^22^^,^^23^

A three-and-a-half-fold improvement in thrombolytic efficiency per milligram of r-tPA administered was observed with the bolus-only protocol (Figure 2A). This may be because more of the administered r-tPA can interact with fibrin-bound plasminogen within the pulmonary arterial thrombus. The endoluminal surface area that is exposed to r-tPA and endogenous urokinase by the repeated expansion and collapse of the infusion basket is much greater than that produced by the passage of a nonexpandable single-lumen infusion catheter. Furthermore, the high-pressure pulse sprays of dilute r-tPA into the clot enable molecules of r-tPA to be trapped in the clot where they can act for several hours, unlike circulating r-tPA, which has a half-life of only 5 to 6 minutes. This increase in r-tPA efficiency is an important advance in managing patients with acute PE, as it can reduce the bleeding risks and treatment costs without compromising efficacy.^9^^,^^10^^,^^16^^,^^17^^,^^24^^,^^25^

The median time required to complete the entire PML procedure was 39 minutes. The dwell time of the Bashir catheter was only 17 minutes, and none of the treated patients required an escalation of care or ICU observation. Returning the patient to a regular floor after the procedure is another significant milestone in CDT treatment, which has important implications for the patient's overall length of stay and hospital resource utilization.^18^

There were no major bleeding events in this study. Only 1 patient developed a clinically relevant nonmajor bleed as a result of an access site ooze (managed successfully with manual compression). The safety profile of this on-the-table approach is encouraging as the rate of major bleeds is still close to 7%, even in contemporary clinical trials of catheter-based thrombectomy or thrombolysis.^18^

### Study limitations

The RESCUE-II was a small feasibility study that included 9 patients. Although several individual operators have reported success in treating patients with acute PE using on-the-table high-pressure pulse sprays of r-tPA without subsequent infusion or ICU stay, the goal of this study was to rigorously evaluate the feasibility and safety of such an approach, using an independent core laboratory. Given that all previously published CDT trials used an infusion protocol, we wanted to ensure that the bolus-only approach provided efficacy and had an acceptable safety profile. Our study did not find any statistically significant change in invasive PA pressures or CO/CI soon after treatment with PML. Importantly, the postprocedural hemodynamics were measured at a median time of 17 minutes after commencing administration of r-tPA boluses. Given that thrombus-bound r-tPA will continue to work on the thrombus for many hours after the initial bolus is administered, we expect ongoing hemodynamic improvement to continue even after the procedure is completed. This is evidenced by the fact that the RV/LV ratio and RMMI scores at 48 hours were both significantly reduced compared with baseline. The final limitation of the single-arm study is the lack of a comparator group like standard CDT infusion or anticoagulation alone.

## Conclusions

Our study demonstrates that treating patients with intermediate-risk PE using on-the-table PML with Bashir catheter is feasible and safe. The high-pressure bolus-only approach resulted in significant improvements in RV/LV ratio, PA obstruction, and reduction in total and subtotal segmental artery occlusions, all achieved with a significantly lower dose of r-tPA compared to conventional infusion protocols. Our approach not only improved thrombolytic efficiency but also avoided an obligatory ICU admission. If expanded to a larger patient population, this novel approach can result in significant cost savings and more efficient use of hospital resources. These promising results warrant further investigation in larger, multicenter trials like the RAPID-PE, which is currently enrolling patients in the United States.

Perspectives **COMPETENCY IN PATIENT CARE AND PROCEDURAL SKILLS:** The present study illustrated that on-the-table PML without postprocedural infusion is a safe and effective treatment option for management of patient with acute intermediate-risk PE. Given that the procedure can be performed quickly and does not require a postprocedural ICU admission, on-the-table protocol can potentially increase the access of this treatment to more patients and allow for a more cost-effective treatment option.

**TRANSLATIONAL OUTLOOK:** This approach should be further evaluated in larger studies such as the RAPID-PE (RESCUE Advanced Protocol without ICU stay and no lytic Drip – for the Treatment of Pulmonary Embolism: The RAPID-PE study) as well as in head-to-head comparisons vs standard CDT, MT, and anticoagulation.

## Funding support and author disclosures

The Department of Community and Economic Development of the Commonwealth of Pennsylvania sponsored the study. Dr Lakhter has received consulting fees/honorarium from Terumo, Thrombolex, Neptune Medical, and Magneto. Dr Firth is an ex officio chief scientific officer and a shareholder in Thrombolex. Dr Bashir is a coinventor of the Bashir endovascular catheter and has equity interest in Thrombolex. All other authors have reported that they have no relationships relevant to the contents of this paper to disclose.
