# Supplementary material for: Safety and Feasibility of On-the-Table Pharmacomechanical Lysis for Acute Intermediate-Risk Pulmonary Embolism: The RESCUE-II Study
Source: JACC Adv. 2025 May 19;4(6):101789. doi: 10.1016/j.jacadv.2025.101789 (PMC12149391; doi:10.1016/j.jacadv.2025.101789)
Supplement: Supplementary data [file mmc1.docx]

| **Supplemental Table 1.** Inclusion and Exclusion study criteria | |
| --- | --- |
| General Inclusion Criteria | |
|  | Willing and able to provide informed consent |
|  | Age 18 years of age to ≤ 75 years of age |
|  | PE symptom duration ≤ 14 days |
|  | Filling defect on at least one main or lobar pulmonary artery as determined on CTA |
|  | RV/LV diameter ratio ≥ 0.9 by CTA as determined by the investigative site |
|  | Willing and able to comply with all study procedures and follow-up |
| General Exclusion Criteria | |
|  | CVA or TIA within one (1) year |
|  | Head trauma, active intracranial, or intraspinal disease ≤ one (1) year prior to inclusion in the study |
|  | Active bleeding from a major organ within one (1) month prior to inclusion in the study |
|  | Intracranial condition(s) that may increase the risk of bleeding (e.g., neoplasms, arteriovenous malformations, or aneurysms) |
|  | Patients with bleeding diatheses |
|  | Hematocrit < 30% |
|  | Platelets < 100,000/μL |
|  | INR > 1.5 if currently on warfarin (Coumadin®) |
|  | aPTT > 50 seconds in the absence of anticoagulants |
|  | Major surgery ≤ 14 days prior to inclusion in the study |
|  | Serum creatinine > 2.0mg/dL |
|  | Clinician deems high-risk for catastrophic bleeding |
|  | History of heparin-induced thrombocytopenia (HIT Syndrome) |
|  | Pregnancy |
|  | SBP < 90 mmHg > 15 minutes within two (2) hours prior to BEC procedure and is not resolved with IV fluids |
|  | Any vasopressor support |
|  | Cardiac arrest (including pulseless electrical activity and asystole) requiring active cardiopulmonary resuscitation (CPR) during this hospitalization at treating institution and/or referring institution |
|  | Evidence of irreversible neurological compromise |
|  | Life expectancy < one (1) year |
|  | Use of thrombolytics or glycoprotein IIb/IIIa inhibitor within 3 days prior to inclusion in the study |
|  | Use of non-vitamin K oral anti-coagulants (NOACs), such as rivaroxaban (Xarelto®), apixaban (Eliquis®) dabigatran (Pradaxa®), edoxaban (Savaysa®) within 48 hours prior to inclusion in the study |
|  | Profound bradycardia requiring a temporary pacemaker and/or inotropic support |
|  | Previous enrollment in this study |
|  | Morbidly obese patient who by the judgement of the investigator is high risk for bleeding |
|  | BMI > 45kg/m2 |
|  | Absolute contraindication to anticoagulation |
|  | Uncontrolled hypertension defined as SBP > 175mmHg and / or DBP > 110mmHg with pharmacotherapy within two (2) hours prior to inclusion in the study |
|  | Subject is currently participating in another study |
|  | Any arterial line placement |
|  | Current positive COVID diagnosis, or ≤ 8 weeks negative of COVID, or > 8 weeks from positive COVID test and with current symptoms, or current active viral pneumonia on chest CT scan |
|  | In the opinion of the investigator, the subject is not a suitable candidate for the study |

| **Supplemental Table 2.** Primary and Secondary endpoints | |
| --- | --- |
| Primary efficacy endpoint | Reduction in the CTA-derived RV/LV ratio at 48 hours after procedural completion |
| Primary safety endpoint | Rate of major bleeds at 72 hours (ISTH criteria)* |
| Secondary endpoints | |
|  | Changes in PA obstruction as defined by the Refined Modified Miller Score (RMMI) as measured on contrast-enhanced chest CTA within 48 hours after the completion of PML |
|  | Reduction in segmental PA total and subtotal occlusions at 48 hours post-PML using the core laboratory assessment of CTA |
|  | All-cause mortality at hospital discharge through 30-day follow-up |
|  | Serious adverse events (SAEs) through 30-day follow-up |
|  | Adverse events (AEs) through 30-day follow-up |
|  | Unanticipated Adverse Device Effect (UADEs) through 30-day follow-up |
|  | Recurrent PE through 30-day follow-up |
|  | Clinically Relevant Non-Major bleeding: any sign or symptom of hemorrhage (e.g. more bleeding than would be expected for a clinical circumstance, including bleeding found by imaging alone) that does not fit the criteria for the ISTH definition of major bleeding but does meet at least one of the following criteria: a. Requiring medical intervention by a healthcare professional. b. Leading to hospitalization or increased level of care. c. Prompting a face to face (i.e., not just a telephone or electronic communication) evaluation |
|  | Technical procedural complications |
|  | Change in Systolic and mean PA pressure after compared to baseline immediately after PML |
|  | Change Cardiac output (CO by Modified Fick calculation) and cardiac index (CI) following pulse spray r-tPA infusion compared to the baseline |
| * ISTH major bleeding in non-surgical patients is defined as having a symptomatic presentation and:   1. Fatal bleeding; and/or 2. Symptomatic bleeding in a critical area or organ, such as intracranial, intraspinal, intraocular, retroperitoneal, intra-articular or pericardial, or intramuscular with compartment syndrome; and/or 3. Bleeding causing a fall in hemoglobin level of 2.0g/dL (1.24mmol/L) or more or leading to transfusion of two or more units of whole blood or red cells. | |
